# Current Therapeutic Use of Metformin During Pregnancy: Maternal Changes, Postnatal Effects and Safety

**DOI:** 10.7759/cureus.18818

**Published:** 2021-10-16

**Authors:** Huma Quadir

**Affiliations:** 1 Internal Medicine/Family Medicine, California Institute of Behavioral Neurosciences & Psychology, Fairfield, USA; 2 Neurology, California Institute of Behavioral Neurosciences & Psychology, Fairfield, USA

**Keywords:** postpartum, oct, insulin, types 2 diabetes, maternal obesity, pcos, gdm, postnatal, pregnancy, metformin

## Abstract

Metformin is one of the most easily available medications for diabetes and has a relatively low cost. It is not only used in diabetes but is also effective in polycystic ovarian syndrome (PCOS) and obesity. Although insulin is the first choice when it comes to treating pregnant women with gestational diabetes mellitus (GDM), metformin has also been debated as a good choice after modification of diet. As metformin passes through the placenta, it is essential to know its consequence of leading to insulin resistance in the fetus as well as the impact on postnatal development. The use of metformin during GDM has raised many trials demonstrating that outcomes from the use of metformin are similar to those achieved with insulin. Follow-up studies were also conducted that assessed the impact on children exposed to metformin in utero. This review highlights the experimental evidence relating to the use of metformin during pregnancy for different conditions, and its impact on the growth and development of offspring.

## Introduction and background

Metformin has exceedingly been one of the most efficient drugs in the treatment of type 2 diabetes mellitus (T2DM). Metformin is also being used for the polycystic ovarian syndrome (PCOS) due to its availability, low cost, and few occasional side effects. However, it has been known that metformin crosses the placenta through a study proving this by evidencing similar quantities of the drug in both maternal and fetal circulation [1].

Metformin is a biguanide taken orally and absorbed mainly in the small intestine via special cation transporters [2]. It routes through the enterohepatic circulation to reach the liver, later distributing into the body cells through transporters which are also present on the placenta, delivering it to the fetal cells. Some studies have reported these transporters to be an organic cation transporter novel type 2 (OCTN2) [3]. This transporter is located at the maternal surface of the placenta. Organic cation transporter 3 (OCT3) has been demonstrated to be even more significant as it was evidenced that pregnant mice without OCT3 showed attenuated fetal metformin exposure [4]. Further, OCT3 expression was noted to be increasing with gestational age in a murine study, with concentrations reaching up to 128-fold at day 19, making it prominent that metformin transfer to fetal tissues increases with the progress of gestation, with being minimum in early pregnancy [5].

**Figure 1:**
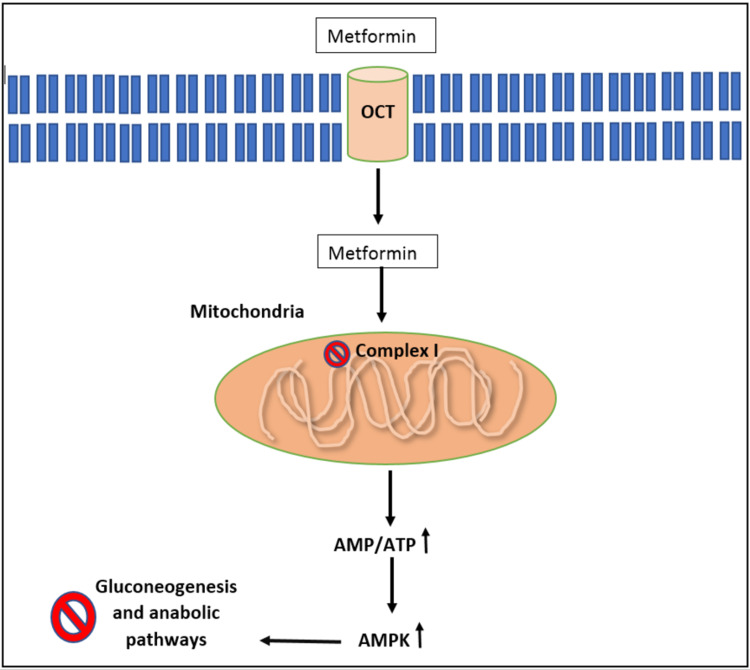
Metformin-induced complex I inhibition. OCT: Organic Cation Transporter; AMP: Adenosine Monophosphate; ATP: Adenosine Triphosphate; AMPK: AMP-activated protein kinase Original diagram

In the mitochondrion, metformin disturbs the electron transport chain by inhibiting complex I, as illustrated in Figure 1, leading to a reduction in adenosine triphosphate (ATP) generation and causing a decrease in the breakdown of adenosine monophosphate (AMP) by blocking AMP deaminase. This leads to an increase in AMP:ATP ratio, and therefore, activates the AMP-activated protein kinase (AMPK). AMPK is a serine/threonine kinase that is required by metformin for its inhibitory effect on glucose production by hepatocytes [6]. Previously, we have known that metformin as a pharmacotherapy, has many uses during pregnancy and that it has also been used to treat gestational diabetes mellitus (GDM). Moreover, its use in maternal obesity and women with PCOS was thoroughly experimented with for efficacy and any associated impacts on gestation. This review will provide details of results concluded by trials for metformin use in the treatment of pregnant women with experimental evidence regarding the long-term effects of metformin in the offspring.

## Review

Use and effects of metformin in gestational diabetes mellitus

The use of metformin is increasing during pregnancy with the incline of obesity and age during pregnancy in our population [7]. Although dietary modification is the primary treatment [8] with insulin being the pharmacotherapy, metformin has achieved a high rate of acceptance as a safe alternative for GDM [9].

A randomized control trial was conducted detailing the use of metformin in women with GDM that included 751 women participants. The participants had GDM and were at different weeks of gestation from 20 to 33 weeks, being treated with the usual therapeutic agent for gravid women, insulin or metformin. A total of 363 women received metformin among which almost 93% stayed on this therapy up until the end of gestation while a few (46%) required the need of insulin in order to achieve better glycemic control. However, it was observed that the need for insulin in that group was still lower than the required insulin dose for the group on insulin alone. This study had results based on the compilation of outcomes including neonatal glycemic state, phototherapy requirement, respiratory distress, an Apgar score of <7 at 5 minutes or premature birth, all of which concluded to have an insignificant difference between the groups. Interestingly, questioning the participants also concluded that 77% of women preferred choosing metformin over insulin for the next pregnancy as compared to only 27% of women preferring it after being on insulin. Further, weight gain was less in women taking metformin than those on insulin. Birthweight and rate of large for gestational age (LGA) were similar in both groups with a lesser rate of hypoglycemia in the metformin group [10]. Although metformin was concluded to have a significant impact on reducing pregnancy-induced hypertension (PIH) versus insulin, it showed no change in preeclampsia [11,12]. A meta-analysis was also conducted which included twenty-one studies, involving 4,545 patients. Subgroup analyses were structured in accordance with different countries of the involved patient as well as treatment strategies applied to the experimental group. It showed statistically significant reduction in gestational hypertension [relative risk (RR) 0.63, 95%CI (0.48, 0.82), P = 0.0006] and maternal hypoglycemia [RR 0.33, 95%CI (0.15, 0.73), P = 0.006] [13].

The use of metformin for GDM has raised many questions on adverse outcomes. While some previous studies have not displayed adverse outcomes from metformin in treating GDM, there are still health departments and associations that have continued to suggest the use of insulin for managing GDM after lifestyle modifications [14,15]. Despite the substantial medical reviews suggesting metformin as a safe choice during pregnancy, long-term safety data are still needed [16].

Metformin use in pregnant obese women

During pregnancy, having obesity associated with high a risk of GDM is likely due to resistance against insulin. This happens due to hormones released by the placenta that have anti-insulin properties. However, obese women have higher insulin resistance as compared to women of normal weight [13]. This causes a high risk of fetal macrosomia, consequently [17]. Data from a randomized control trial including 843 pregnant women with a body mass index of more than 30 kg/m^2^, showed that metformin caused a significant reduction in maternal gestational weight compared to placebo [18]. However, administration of metformin was reported to be accompanied by gastrointestinal side effects including nausea, vomiting, and diarrhea [17].

Another animal study was conducted on eight-week-old male and female mouse offsprings to observe and investigate metformin effects as an intervention during obese glucose-intolerant pregnancy. The effect was mainly investigated on the gonadal white adipose tissue of the offspring. Metformin administered during gestation diminished the adiposity in obese dams and augmented the duration of gestation. The control or female mice on an obesogenic diet (not on metformin) had a similar body weight as the offspring of obese dams that were administered 300mg/kg/d of metformin. Despite the similar weight, young adulthood of both sexes showed adipocyte hypertrophy [19].

Another observation notes that although metformin attenuates the risk of neonatal intensive care unit admissions, it does not have clear evidence of reducing the incidence of large for gestational age (LGA) newborns [20]. Therefore, the advantageous properties of metformin and its impact on the health of neonate and pregnant women when used in gestational obesity are still under debate and its prescription should be cautious.

Use of metformin during pregnancy for the polycystic ovarian syndrome

PCOS is an endocrine abnormality that has gained a very commonplace condition in the list of endocrine disorders. PCOS causes anovulation and is associated with hyperandrogenism causing hirsutism. Further, it causes insulin resistance which on its own has substantial risks on health [21]. Some also state that PCOS may not always present with clinically evident features of androgen excess but will display features of ovarian dysfunction. Both obese and nonobese women with PCOS, insulin resistance becomes more evidently common [22]. It was also suggested that increased risk of obstetric and neonatal complications, PIH, GDM, premature delivery risk, and preeclampsia are due to the pathophysiology involved in PCOS including hyperandrogenism and insulin resistance [23].

For the management of PCOS, metformin has achieved the role of standard pharmacotherapy in improving the metabolic relations of PCOS in glucose intolerance, induction of ovulation, and depressing the miscarriage rates [24]. For possible teratogenic effects, a meta-analysis was constructed around 351 women with first trimester exposures to metformin. It was found to have no increase in birth defects [25].

A study also compared 200 women who had PCOS and were nondiabetic, conceiving while on metformin. They continued the dose of 1-2g/day during pregnancy, with 160 similar cases of women but with discontinuation of metformin on acknowledging the pregnancy. It was evidenced that there was a statistically significant reduction in preeclampsia and GDM in women who continued to take metformin throughout the pregnancy [26]. Further investigating this, another study was carried which analyzed the continued use of metformin throughout pregnancy and showed resulting outcomes of a lower rate of fetal growth restriction, GDM, premature delivery and increase in live births [27]. The study did not report evidence of stillbirths or congenital birth defects. Metformin was also evidenced to be an effective treatment in ovulation induction when combined with clomiphene. This meta-analysis included randomized control trials which compared the results with those on clomiphene alone [28].

More studies were conducted assessing weight in women receiving metformin in pregnancy for PCOS and the weight of their infants one year postpartum. It was noted that women receiving 2g of metformin daily from the beginning of pregnancy to delivery, had lost less weight, with their infants being heavier than those in the placebo group, measured one year postpartum [29]. Further, studies noted that exposure to metformin in utero led to differences in female infants that were not a feature observed in boys. It was observed that one-year-old females had a larger head circumference when compared with those not exposed to metformin in utero. However, both males and females maintained a higher weight at four years of age with an odds ratio of 2.17 (1.04-4.61) to be overweight/obese [30].

Follow-up investigations on metformin exposure in utero

Unregulated glycemic control in diabetes during pregnancy exposes the fetus to high levels of glucose and leads to an increased risk of diabetes later in life, above any risk attributable to genetic factors [31]. Observing effects on infants at different ages on follow-up, investigators found that at two years of age, infants who were exposed to metformin in utero had an increase in subcutaneous fat that was assessed by measuring their subscapular and biceps skin folds. This was in comparison with infants who had no exposure, while the total body fat remained the same for both groups [32].

Twelve children who were exposed in utero to metformin due to maternal PCOS were followed up after eight years and showed increasing fasting blood glucose levels, systolic blood pressure, and low levels of low-density lipoproteins as compared to the unexposed group [33]. Since the sample size was small for this study, its significance is low. Follow-up of similar studies at nine years on metformin-exposed children showed increased weight along with increased arm and waist circumferences [34].

Safety profile

Metformin-associated adverse effects include nausea, vomiting, diarrhea, and mild abdominal discomfort for which reason the usual dose at which it is started for pregnant women is 0.5 g/day and increased over time up to 3 g/day. It is excreted by the kidney, so, proper renal function is required to prevent its retention. Therefore, such patients require monitoring of kidney function tests regularly [35].

A retrospective study was conducted in India to observe the effects of metformin given to women in the first trimester for GDM. It was observed to have no significant adverse outcomes on fetal or maternal health [36]. More recently, a follow-up study was done to assess evidence of increased cardiovascular risk in children with exposure to metformin in utero taken for maternal obesity. It was compared with a group of children born to mothers who participated in a placebo during pregnancy. The results found no evidence that metformin had an impact on increasing cardiovascular risk in those children measured with blood pressure, arterial wave velocity, body composition, and central hemodynamics [37].

A cohort was constructed including women of age 20 to 44 years with type 2 diabetes in Taiwan that had an insulin group, a metformin group, and a group that switched from metformin to insulin. It was concluded that the metformin group had a lower risk of primary cesarean section and congenital malformations while the insulin group and the group that switched to insulin had similar pregnancy outcomes between them that included risk of cesarean section, pregnancy-induced hypertension, and preterm birth [38]. Metformin has been stated to be under thorough assessment for its role as an adjuvant to cancer therapy [39]. Although current studies have not indicated the negative effects of metformin on maternal and fetal outcomes, its exposure still needs attention focusing on its impact on functional growth, neurological development, and reproductive function after long-term exposure in follow-up studies. Metformin should be used with caution during pregnancy.

Limitation

Long-term effects on children with mothers taking metformin have not been thoroughly assessed. This review did not cover the cardiovascular risks in women taking metformin during gestation.

## Conclusions

It is still a question whether metformin can replace insulin as first-line therapy in pregnant women for GDM. Many of the above-reviewed trials concluded metformin to be a safe choice at the beginning of pregnancy without persuasive evidence of increased risk for miscarriages or congenital malformations. An enhanced overview is needed to focus on possible complications that can occur on discontinuation of metformin after being taken for type 2 diabetes and continued to early days of pregnancy. Since metformin has been observed to reduce weight gain in pregnant women, it is the preferred choice in obese women with GDM. Metformin is beneficial in assessing gestational diabetes, obesity, and symptoms of PCOS, but its effects in the long term, particularly post-natal effects, have not been well studied in the population, which pose a need for follow-up studies
